# Effects of Paclitaxel on EGFR Endocytic Trafficking Revealed Using Quantum Dot Tracking in Single Cells

**DOI:** 10.1371/journal.pone.0045465

**Published:** 2012-09-20

**Authors:** Hui Li, Zhao-Wen Duan, Ping Xie, Yu-Ru Liu, Wei-Chi Wang, Shuo-Xing Dou, Peng-Ye Wang

**Affiliations:** Key Laboratory of Soft Matter Physics, Beijing National Laboratory for Condensed Matter Physics, Institute of Physics, Chinese Academy of Sciences, Beijing, China; Technische Universitaet Muenchen, Germany

## Abstract

Paclitaxel (PTX), a chemotherapeutic drug, affects microtubule dynamics and influences endocytic trafficking. However, the mechanism and the dynamics of altered endocytic trafficking by paclitaxel treatment in single living cells still remain elusive. By labeling quantum dots (QDs) to the epidermal growth factor (EGF), we continuously tracked the endocytosis and post-endocytic trafficking of EGF receptors (EGFRs) in A549 cells for a long time interval. A single-cell analysis method was introduced to quantitatively study the dynamics of endocytic trafficking. Compared with the control cells, the velocity of directed motion was reduced by 30% due to the suppression of high speed movements of EGF-QDs along the microtubules in PTX-treated cells. The endocytic trafficking in PTX-treated cells was mainly via super-diffusive mode of motion, whereas in control cells, it was mostly via sub-diffusive mode of motion. Moreover, PTX shortened endosomal trafficking and prevented EGF-QDs from moving to the perinuclear area via the rapid delivery of EGF-QDs into the peripheral lysosomes. The present study may shed light on the mechanism of the effect of PTX on the treatment of lung cancer.

## Introduction

Endocytosis and post-endocytic trafficking of surface-expressed receptor proteins are complex dynamic processes for eukaryotic cells. These processes are also critical throughout the whole cellular signaling, including receptor internalization, endosomal trafficking, and lysosomal degradation or recycling to the plasma membrane [1], [2], [3], [4]. The epidermal growth factor receptor (EGFR) endocytosis is one of the best characterized models for studying the mechanism, kinetics, and route of endocytic process, as well as other receptor tyrosine kinases [5], [6], [7], [8]. Recently, our understanding of receptor endocytic trafficking has been greatly advanced by new imaging techniques, especially real-time imaging in living cells [9], [10], [11], [12], [13]. Quantum dots (QDs), as novel fluorescent probes with bright fluorescence and excellent photostability, are participating more in single-molecule imaging experiments of endocytic trafficking because QDs, functionalized with receptor ligands, provide the means to activate membrane receptors and to track their endocytic pathway directly in living cells with high sensitivity and long duration [14], [15].

QDs bearing ligands, such as the EGF [16], [17] and nerve growth factor [18], [19], constitute an exquisitely sensitive tool to explore the dynamic behavior of molecules in endocytosis. Some details about the internalization mechanism and dynamic information of growth factor receptors have been revealed, including receptor heterodimerization, endosomal transport rate, and retrograde transport. These previous studies facilitated the measurement of endosomal trafficking and provided closer insights into the endocytic pathway. However, they focused mainly on the local feature of target endocytic receptors in several seconds or in a few minutes, which may limit the global descriptions of the endocytic process in more than 30 min in living cells [1], [3]. Moreover, the statistical results obtained from repeated single-molecule experiments have blurred the correlation between the measured dynamic information and the time-dependent behavior of endocytic process, making the data unreliable and difficult to interpret. Thus, studying endocytosis and post-endocytic trafficking by tracking a large number of endocytic receptors simultaneously in a single cell throughout the receptor’s lifetime is necessary. A new data processing method is also required to analyze original raw data from QD-labeled receptors and to quantify the endocytic process at a single-cell level. The capability of studying single cells will bring better understanding of cellular heterogeneity.

Paclitaxel (PTX), a microtubule (MT) stabilizing drug, is a widely used chemotherapeutic agent in many types of cancers, including lung cancer, ovarian cancer, breast cancer, as well as other types of solid tumor cancers [20], [21], [22], [23]. The important effect of PTX is to suppress MT dynamics and block mitosis, inducing apoptotic cell death [24], [25], [26], [27]. Moreover, PTX has other diverse effects on endocytic trafficking, such as disruption of membrane trafficking [28], [29], [30] and change of signal transduction [31], [32]. These effects are important and also require further study because of the significant role played by endocytosis in human cancer [33], [34]. However, due to lack of direct and integrated methods to quantify the dynamic behavior of the endocytic process, the precise mechanism and dynamics of altered endocytic trafficking by PTX treatment in a single living cell still remain largely unknown.

In the present study, labeled EGF-QDs were used to track the endocytosis and post-endocytic trafficking of EGFRs continuously over a long interval in lung carcinoma A549 cells. A single-cell analysis method was introduced to quantitatively study the dynamics of endocytosis during the first 5 min period by examining the fluorescent intensity of EGF-QDs. The dynamics of endocytic trafficking during the following 60 min period was also determined by analyzing the trajectories of EGF-QDs. Results showed that in PTX-treated cells, the mean velocity of the directed motion of endocytic trafficking decreased by about 30%, and the diffusion of endocytic trafficking showed a more active behavior compared with the control cells. To further investigate the destination of endocytic trafficking in PTX-treated cells, immuno-colocalization assays were performed to identify the compartments where the endocytic complexes reside. Through colocalization analysis of EGF-QDs in early endosomes and lysosomes, we found a fast lysosomal delivery of EGF-QDs in PTX-treated cells, revealing that PTX shortened the endosomal trafficking by the peripherally distributed lysosomes. Based on our results, we proposed a model for EGFR endocytic trafficking under PTX treatment.

## Results and Discussion

### Internalization and Fusion of EGF-QDs were Not Affected by PTX

**Figure 1:**
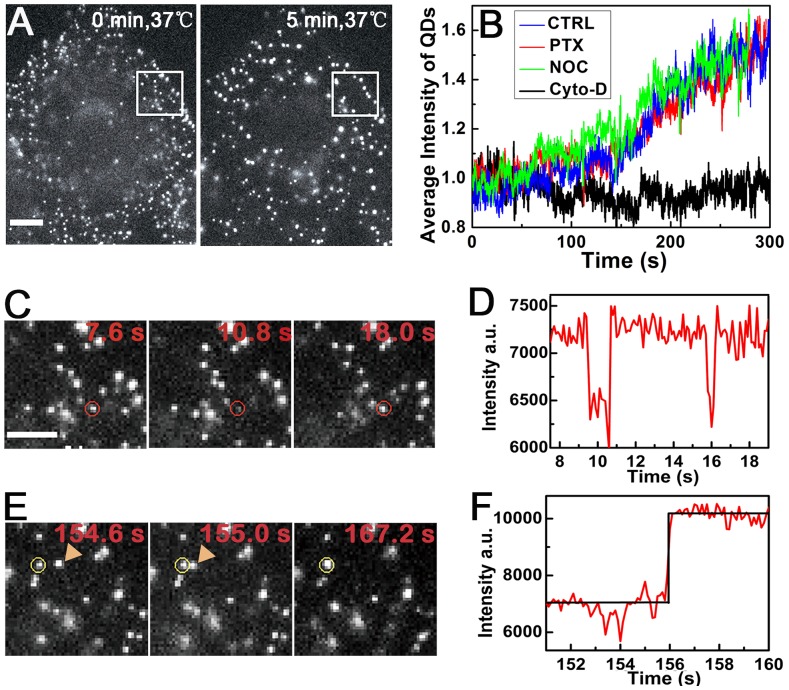
EGF-QD binding, internalization, and aggregation during the first 5 min of endocytosis. (A) A549 cells were treated with 100 nM PTX for 4 h prior to labeling. The surface EGFRs were labeled with EGF-QDs at 4°C, and internalized after heated up to 37°C. The images **s**howed typical aggregation of QDs in PTX-treated cells during the first 5 min. Scale bar: 10 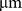. (B) The normalized average intensities of the fluorescence puncta in the control (CTRL), PTX-treated, NOC-treated, and cyto-D-treated single cells. Increases in fluorescence intensity indicate aggregation of EGF-QDs in endosomes. The concentration of NOC used is 60 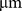 and that of cyto-D is 20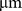. (C, E) Magnified image of QDs indicated in panel A. Panel C: blinking of a single QD particle (*red circle*); panel E: one QD (*arrow*) moved fast toward the left QD (*yellow circle*), and fused together. See Movie S1. (D, F) The time traces of fluorescence intensity of the QDs (*in circle*) in panels C and E, respectively.

We first studied the dynamics of EGFR endocytosis during the first 5 min by analyzing the temporal evolution of the fluorescent intensity of EGF-QDs. A549 cells were treated with 100 nM PTX for 4 h prior to the experiment [24], [35]. These conditions have been proven effective in inhibiting cell proliferation. The EGFRs on the cell surface were labeled with EGF and QDs using the consecutive binding method previously described [16], [36], [37], [38]. EGF does not significantly dissociate from EGFR unless in the acidic environment of lysosomes [39], [40], so the EGF-QD is denoted as the EGFR-EGF-QD complex in endocytosis and subsequent intracellular trafficking in the cytoplasm. By adjusting the temperature of the cells, the starting moment of endocytosis can be controlled precisely in the experiments. After the PTX-treated cells were heated up to 37°C, single-blinking EGF-QDs immediately emerged on the cell membrane, agglomerated, and then formed non-blinking aggregates with higher intensity (Figures 1A and 1B). This result indicates that the activated EGFRs internalized and fused with other endocytic vesicles (EVs) that contained EGFR-EGF-QDs during the first 5 min of endocytosis, consistent with previous data [6], [41]. The sequence images show the typical characteristics of single-blinking EGF-QD (*red circle*) at the initial internalization (Figure 1C) and that of the vesicular fusion process, during which the EV (*arrow*) moved fast toward the left EGF-QD (*yellow circle*) and fused to it irreversibly (Figure 1E and Movie S1). The two characteristics can be clearly seen in Figures 1D and 1F, where the measured fluorescence intensities of the EGF-QDs are shown for the two cases. To study endocytosis globally, we tracked almost all EGF-QDs simultaneously in single cells and calculated the average intensity of the puncta (single or aggregated QDs) in each frame during the first 5 min (Figure 1B). The fusion process is mainly characterized by increasing the intensities of puncta, so it is practical to quantify endocytosis during this period by analyzing the intensity of QDs rather than their numbers or positions. To further verify the increase in fluorescence intensities measured under our platform, we used a low N.A. objective lens to take the entire wide-field images. The same result was obtained, confirming the quantization of the acquired signals (Figure S1).

During this period, the characteristics observed in PTX-treated cells were similar to those observed in control cells (Figure 1B), probably because PTX, as the MT-stabilizing agent, does not interfere with the actin-dependent EV activity during internalization, such as transport and fusion of EVs [42]. To prove this supposition, we incubated cells with cytochalasin-D (cyto-D) that disrupted actin filaments. The measured intensity of QDs remained nearly at the same level during the first 5 min (Figure 1B), indicating that cyto-D treatment was likely to inhibit the fusion and transport of newly formed EVs by disrupting actin filaments in A549 cells, consistent with previous observations [43]. In addition, nocodazole (NOC), as the MT-disrupting drug, was also tested and found to exert no disturbance on the fusion of newly formed EVs in A549 cells (Figure 1B), further proving that MT-targeted drugs, such as PTX, have no effect on receptor internalization. Moreover, based from the similar increases in fluorescence signals, there is no difference in EV trafficking between MT stabilization and disruption during the first 5 min.

### Tracking EGF-QDs in Single Living Cells Revealed that PTX Reduces Endocytic Ratios of EVs

We subsequently studied the dynamics of endocytic trafficking after the early stage of endocytosis by tracking all the EGF-QDs for 60 min in single cells. We performed continuous imaging focused on one layer of the cell to study the whole endocytic trafficking process (Figure S2). For convenience of writing, we used *t = *0 min as the moment when the early stage of endocytosis was completed, i.e., at 5 min since the initiation of internalization of EGFRs.

**Figure 2:**
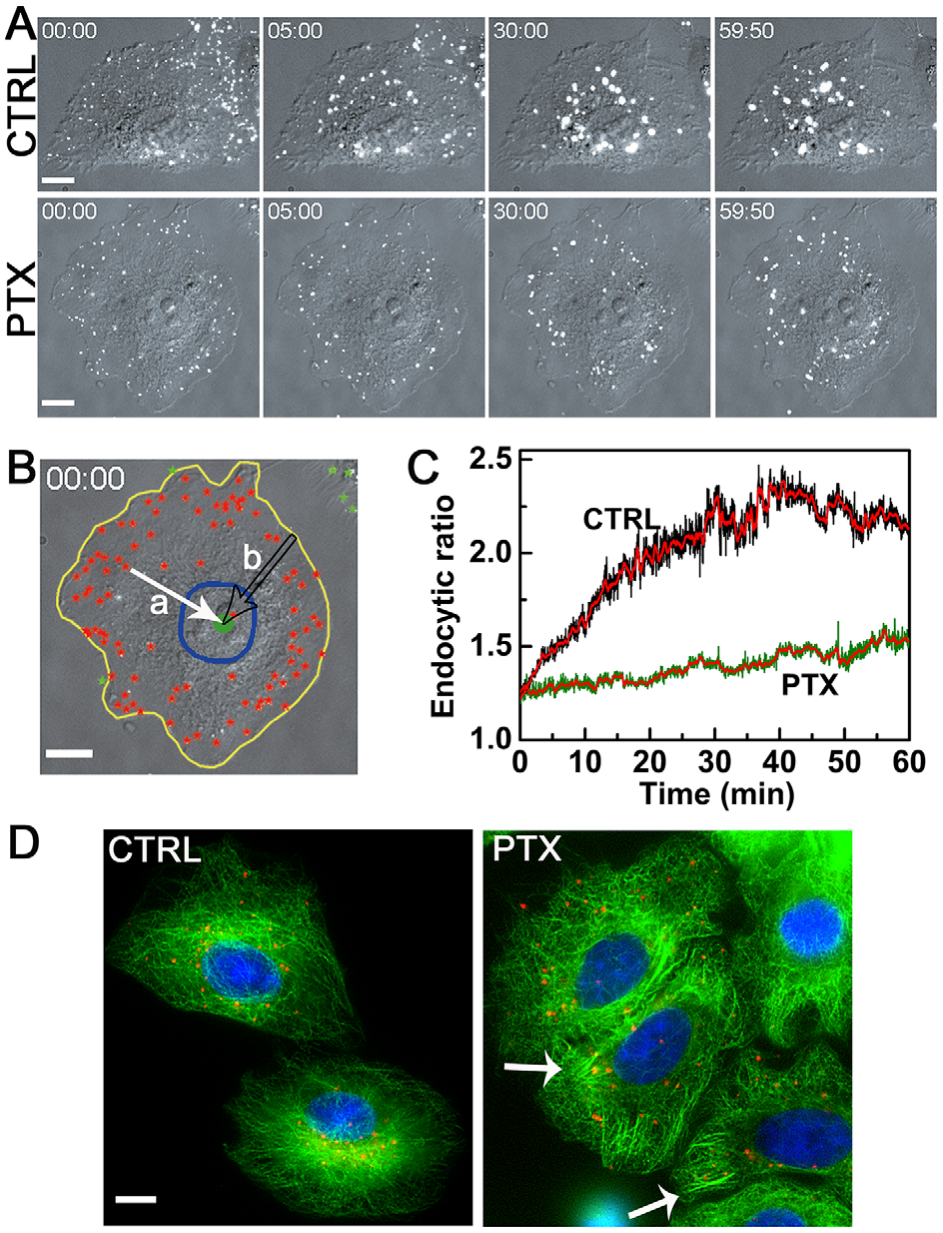
PTX restricts endocytic transports near the periphery of the cytoplasm. (A) The time-lapse images of endocytic trafficking in A549 cells at *t* = 0, 5, 30, 60 min (time resolution: 100 ms, time stamp in min: sec). The fluorescent images of the QDs are overlaid with the bright-field DIC images of the cell. Endocytic vesicles moved toward the center of the cell and fused with other EVs continuously when traveling in the cytoplasm in the control cells. The EVs in the PTX-treated cells remained near the periphery and fused with other EVs in a minor fashion. The concentration of PTX used is 100 nM. See Movies S2 and S3. (B) Illustration of the endocytic ratio calculation. The intracellular QDs are marked by red stars, whereas extracellular ones are marked by green stars. The boundaries and nuclear of the cell are highlighted in yellow and blue lines, respectively. The center of the cell is marked by a green dot in the nucleus. All the sites are selected in DIC images. The change of the cell shapes in 60 min is negligible, as examined from the DIC images obtained before and after every 30 min fluorescence imaging. The distance of QDs from the center of the cell is denoted by the solid arrow ‘a’, and the distance of the corresponding points on the cell boundaries to the center of the cell is denoted by the hollow arrow ‘b’. The endocytic ratio equals the mean value of b/a. (C) The endocytic ratio of the control and PTX-treated cells in panel A is a function of time. Red lines represent the smooth data obtained by averaging 20 adjacent points along the original lines. (D) The merged fluorescent images of QD (*red*) and MTs (*green*) after 60 min of EGF-QD endocytosis under the identical condition to panel A. Cells were labeled with rat anti-Tubulin and Alexa-488 goat anti-rat IgG conjugate. The nucleus was stained with DAPI (*blue*). All scale bars: 10 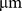.

After internalization, EGF-QDs were transported in both control and PTX-treated A549 cells, and the fluorescent images of the QDs were overlaid with the bright-field DIC images of the cell (Figure 2A and Movies S2 and S3). The EVs in both control and PTX-treated cells moved rapidly inward during the first 5 min. The EVs in the control cells were more aggregated and moved fast toward the cell center after *t = *5 min. Almost all EGF-QDs aggregated near the nucleus at *t* = 30 min and 60 min. However, in PTX-treated cells, the EVs did not exhibit inward movement and only moved near the periphery of the cell. Even at *t* = 60 min, only a few EVs reached the periphery of the nucleus. A method to quantify the two different endocytic trafficking behaviors was developed. The method is characterized by the endocytic ratio, which is defined as the ratio of the average radius of the cell (distance of points on cell boundaries to the center of the cell) to the average distance of all the EVs to the center of the cell in each frame (Figure 2B). The obtained endocytic ratios versus time in the control and PTX-treated cells are shown in Figure 2C. In the control cells, the rapid increase of the endocytic ratio indicated that the endosomal compartments underwent a fast transport from the periphery to the center of the cell. However, the transport in PTX-treated cells was slow, with an almost invariable velocity. The two curves started at nearly the same position of 1.2 at *t* = 0 min, became wider with time, and were maximized at *t* = 40 min because at this time, EVs in the control cells had reached their destinations, which was near the periphery of the nucleus. These results clearly demonstrated that endocytic trafficking can be seriously hindered by PTX and most EVs end near the periphery of the cell, consistent with previous data [29]. Moreover, the obtained endocytic ratio in NOC-treated cells was similar to that in PTX-treated cells (Figure S3), implying that the endocytic ratio could be changed by either stabilizing or depolymerizing MTs.

To investigate the effect of MTs on the transport of EVs, the cells were fixed and subjected to fluorescent staining immediately after the experiments. Figure 2D shows that the MTs in the PTX-treated cells formed bundles that appeared near the periphery of the cells (arrow), consistent with previous reports [35], whereas in the control cells, the MTs were oriented from the MT organization center located near the nucleus. A previous study has reported that in PTX-treated cells, the 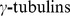 are not associated with the peripherally aligned MTs, nor located near the nucleus, resulting from the suppressed MT dynamic [44]. The origin of the redistributed MT network by PTX is not fully understood up to now. The endocytic EGF-QDs in PTX-treated cells were scattered near the periphery of the cells, whereas in the control cells, they were located near the nucleus (Figure 2D). The percent of the cells showing EGF-QDs in the periphery of the PTX-treated cells was about 86.7% (of total 83 cells), whereas it was only 6.6% (of total 91 cells) in the control cells. The restricted endocytic transport was not solely due to the rearrangement of MTs by PTX treatment. There were still some unaffected MTs in the PTX-treated cells that could serve as the track for EVs to transport to the center of the cells, as in the control cells. The potential origin of the peripheral distribution of EVs by PTX treatment would be studied using immunofluorescence approach.

### PTX Reduced the Velocities of Directed Motion of EVs on MTs but Enhanced the Proportion of Super-diffusion

**Figure 3:**
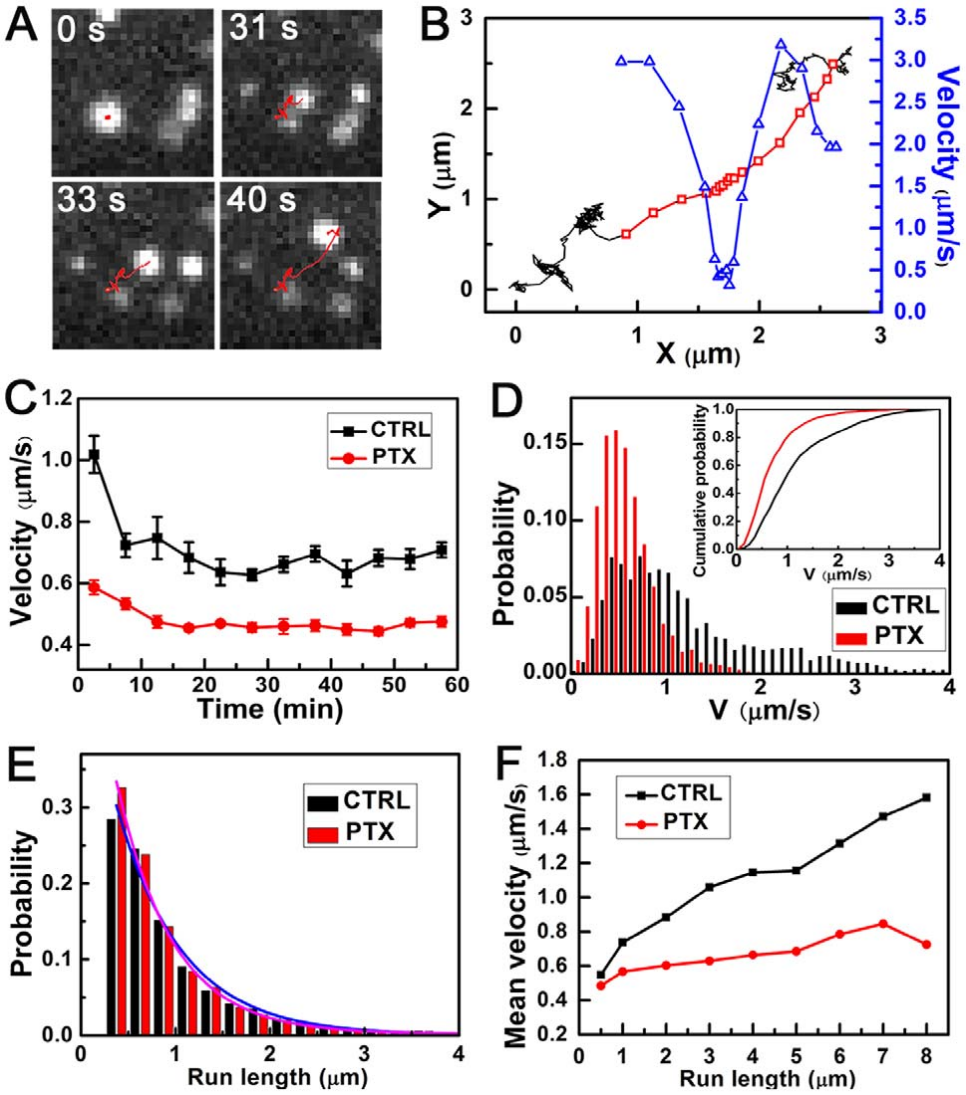
Dynamic analysis of directed motion of EVs on MTs. (A) Tracking of the typical EV in a part of the cell, with the path marked by red line. See also Movie S4. (B) Trajectory (start at *x* = 0, *y* = 0) of the EV shown in panel A is segmented into directed motion (*red*) and non-directed motion (*black*). Instantaneous velocity versus *x* in directed motion is marked by blue triangles. (C) The mean velocities of the directed motion during each period of 5 min in both control and PTX-treated cells. (D) Typical probability distributions of velocities during time period *t* = 0 min to 5 min. Inset: Cumulative distributions for control (*black*) and PTX-treated (*red*) data. The data were from one control cell (velocity sample number: *n* = 3318) and one PTX-treated cell (*n* = 10071). The data during *t* = 35 min to 40 min are shown in Figure S5. (E) Distributions of the run lengths in control and PTX-treated cells. (F) The mean velocities of the directed segments versus the given run lengths in the control and PTX-treated cells. CTRL (6 cells, total sample *n* = 6335), PTX (8 cells, *n* = 19890). Data are presented as mean ± SE. The total sample size is shown in Table S1. The concentration of PTX used is 100 nM.

To further investigate the endocytic dynamics, more than 2,000 EGF-QD trajectories in each cell during the 60 min period were collected and analyzed (Table S1). The dynamic parameters of the EGF-QD motion were calculated. In general, the trajectories were composed of one or several phases of directed motion separated by periods of non-directed motion (Figure 3A).

On the directed motion, we used an automatic identification method to extract the directed segments of the whole trajectories. The method is based on the analysis of the mean square displacement (MSD) and the directional persistence, as previously done (for details see Materials and Methods) [45]. Prior to our analysis, we performed two verification experiments. First, we showed that after disrupting the MTs by NOC, the directed segments were almost completely suppressed. This finding indicated that the obtained directed motion is MT-dependent, without any actin-based movements (Table S1). Second, we used a published identification method based on support vector machine (SVM) to analyze the data and compare the results with ours. The same measured velocities proved the reliability of our method (Figure S4).

An example of EGF-QD trajectories inside A549 cells is displayed in Figure 3A and Movie S4, with the phase of directed motion and the corresponding moving velocity of each point plotted by red and blue lines, respectively (Figure 3B). Figure 3C shows the mean velocities of the directed motion during each period of 5 min interval in both control and PTX-treated cells. The velocity in the PTX-treated cells (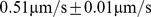) decreased by about 30% (about 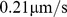) compared with that in control cells (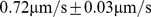). The measured velocities in control cells are consistent with the previous studies of vesicular movements [13], [46]. The distributions of the velocity revealed that the directed motion with higher velocities was suppressed by PTX treatment (Figures 3D and S5). However, only a slight difference was observed in the run length, as shown in Figure 3E. The control cells had a mean run length of 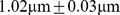, as previously reported [47], whereas the PTX-treated cells had a mean run length of 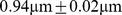, which is only about 7.8% shorter than the control cells (Table S1). In other words, the mean run length or mean stepping number of the motor on MTs was nearly not reduced by PTX treatment, implying that the affinity of the motor proteins for MTs was almost unaffected by PTX treatment. The relationship between the mean velocities and the run length of each directed segment was also studied. The mean velocities increased with the run length in the control cells, consistent with previous observations [48], [49]. By contrast, the mean velocities did not increase with run length in the PTX-treated cells (Figure 3F).

PTX could affect the polymerization dynamics of MTs [24], which in turn induces the motion of MTs in cells. From previous data, the presence of 100 nM PTX could change the rates of growth and shorting of MTs by about 0.05 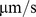 [35], which is much smaller than the moving velocity difference of about 0.21 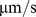 measured in this study. Thus, we concluded that the velocity difference of the directed motion in the control and PTX-treated cells mainly resulted from the velocity difference of the motor proteins moving along MTs, whereas the growth and shorting of MTs have little effect on the velocity difference. On the other hand, PTX binds directly to the tubulin and presumably induces the conformational change of the 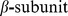, along the length of the MT with a binding site on the 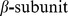 [50]. Therefore, our results implied that the conformational changes of the MTs could affect the velocity of the motors moving along the MTs while having little effect on the overall affinity of the motors for MTs. The decrease in the velocity by PTX treatment could either result from the decrease in the MT-mediated ATP-hydrolysis rate [51] or the mechanochemical coupling efficiency, both induced by MT conformational change [52].

**Figure 4:**
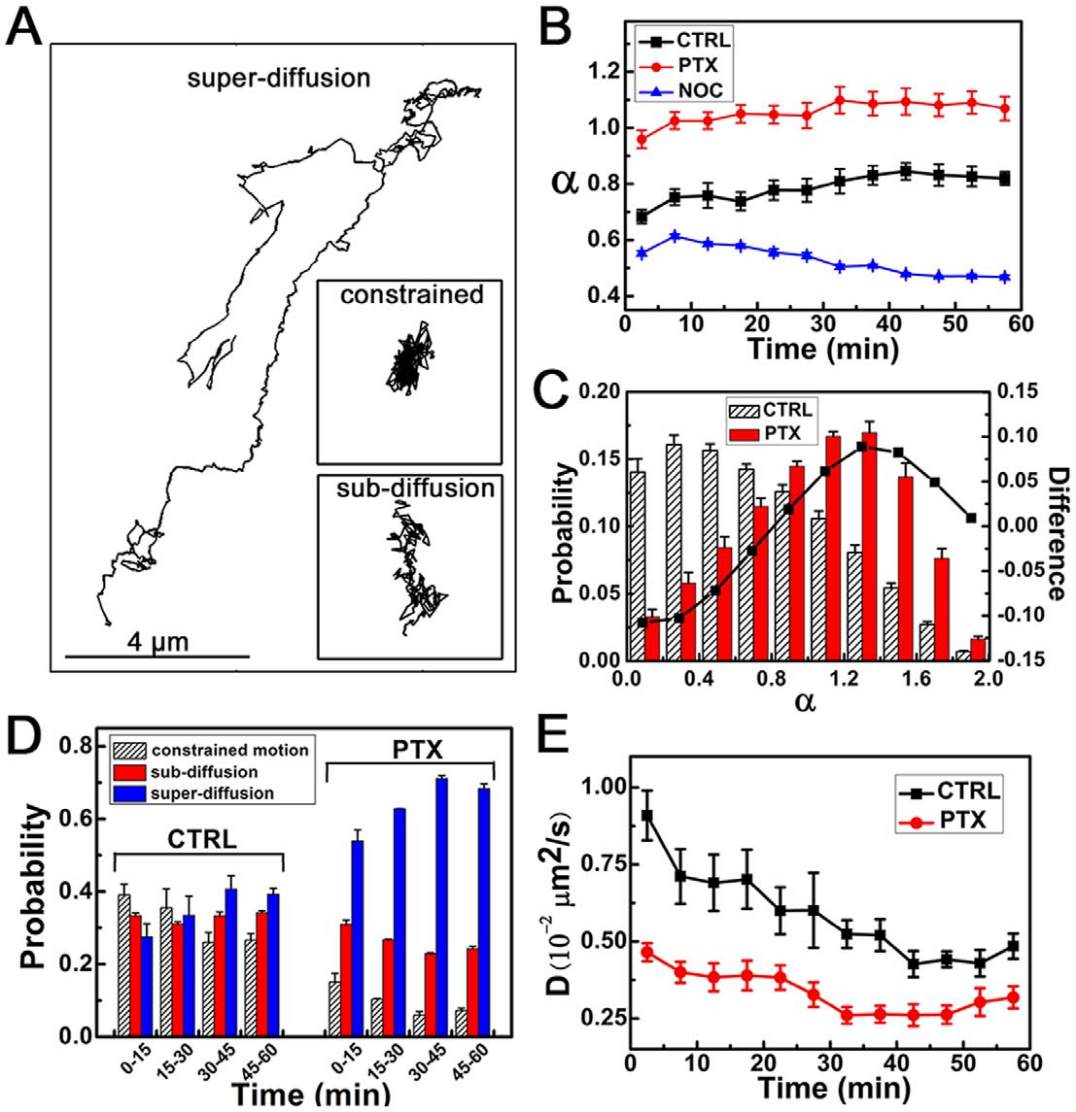
Dynamic analysis of total motility. (A) Selected EGF-QD trajectories: constrained motion (with mean 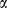 as 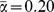), sub-diffusive motion (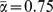), and super-diffusive motion (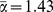). Scale bar: 4 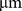. (B) The mean 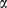 of the total motility during each period of 5 min interval in control, PTX-treated, and NOC-treated cells. (C) Probability distributions of 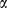 during *t* = 5 min to 10 min in control and PTX-treated cells. The solid line represents the difference in the values of 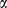 between the two cases in each bin. The data during *t* = 45 min to 50 min is shown in Figure S7. (D) Probability of constrained motion, sub-diffusion, and super-diffusion during each 15 min interval (i.e., 0 min to 15 min, 15 min to 30 min, 30 min to 45 min, and 45 min to 60 min) in control and PTX-treated cells. (E) The mean diffusion constant, *D*, of the sub-diffusion during each interval of 5 min in both control and PTX-treated cells. The data were analyzed as described in Figure 3. Data are presented as mean 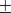 SE. The sample size is shown in Table S1. The concentration of PTX used is 100 nM and that of NOC is 60 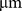.

The proportion of directed motion in all the trajectories was below 3%, whereas most of the trajectories belonged to the non-directed motion (Table S1). The trajectories of the non-directed motion also contained some transient directed movements that could not be identified when their duration was shorter than the threshold (five consecutive frames). To provide a systematic, unbiased description of total motility, we applied time-resolved MSD analysis to quantitatively study the whole trajectories. For each point of the trajectory, the local MSD was fitted by 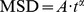. 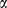 indicates the non-linear relationship of MSD with time and carries information about the local motion modes [53], [54]. Free Brownian motion of the intracellular vesicles is highly restricted in the crowded cytoplasm in living cells [55]. Therefore, we classified the movements of EVs into three modes of motion according to the value of 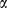: constrained motion (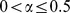), sub-diffusion (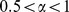), and super-diffusion (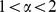) (Figures 4A and S6, see Materials and Methods for details). During sub-diffusion, the respective mean 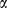 of the control and PTX-treated cells were 0.73 and 0.75, consistent with the previous measured values of sub-diffusion [56]. To compare the global feature of the EV motility in PTX-treated cells with that of control cells, the mean values of 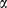 of all the trajectories in both cells during each period of 5 min interval are shown in Figure 4B. The motion of EVs in PTX-treated cells was characterized by larger values of 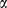 (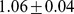) compared with the control cells (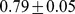). Moreover, most of the movements in PTX-treated cells were super-diffusive motion, whereas in control cells, most of the movements were constrained and sub-diffusive motions (Figures 4C and S7). As time increased, the proportion of constrained and sub-diffusive motion (with 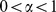) decreased, whereas that of super-diffusion (with 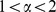) increased (Figure 4D). The motion in the super-diffusion mode is diffusion overlaid with the directed motion, with the former having no association with MT-dependent trafficking and the latter associated with MT-dependent trafficking. This finding could be seen by comparing the values of 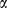 in the control and NOC-treated cells in Figure 4B, where the motion in NOC-treated cells has an even smaller 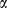 (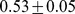) than the control cells. Thus, there were more MT-dependent movements in the total motility of the PTX-treated cells, inducing the increased proportion of super-diffusion. This phenomenon can also be noted from the proportion of directed motion that increased from 1.28% in the control cells to 2.99% in the PTX-treated cells (Table S1). Considering that MTs were aggregated near the periphery of the PTX-treated cells (Figure 2D), the increased proportion of super-diffusion by PTX treatment presumably resulted from the increased probability of the EVs to bind to MTs and jump between different MTs. However, in the control cells, the MTs were oriented from an MT organization center located near the nucleus, so the density of MTs near the periphery of a cell is low, whereas it is high near the nucleus. Thus, the EVs showed low probability to bind to MTs near the periphery of the cells, resulting in a low proportion of directed motion on MTs near the periphery in the super-diffusion mode. However, near the nucleus, the probability for the EVs to bind to MTs and jump between different MTs increased, resulting in the increase of the proportion of directed motion in the super-diffusion mode.

Diffusion constant *D* was determined by fitting the initial 5 points of the MSD curve with 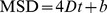
(dashed lines in Figure S6) [37]. The diffusion constants of sub-diffusive motion (with 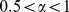) in PTX-treated cells were approximately 40% lower than those in control cells during the 60 min period (Figure 4E). One possibility is that PTX induced the formation of MT bundles near the cell periphery (Figure 2D), which could serve as an effective boundary, resulting in the lower diffusion constant. This finding is consistent with the observations of Faucheux and Libchaber [57], showing that the diffusion constant of a particle was reduced when it approached the boundary. The other possibility is that intracellular components became more crowded near the peripherally distributed MTs in PTX-treated cells, resulting to higher possibilities for EVs to collide with the intracellular components. Thus, the diffusion constants of EVs are reduced, as reviewed by Dix and Verkman [54] that the molecular crowding is the major determinant in the reduction of macromolecule diffusion.

The above results provided a global description of the effects of PTX on endocytic trafficking dynamics. Given that endocytic trafficking is correlated with the endocytic route, we propose that the EVs under PTX treatment undergo transports along paths that are different from the EVs in the control cells, and they possibly end in compartments involved in the endocytic transport (see below).

### Immuno-colocalization Showed that PTX Shortens Endosomal Trafficking

To study the origin of restricted endocytic trafficking induced by PTX treatment, we aimed to identify the compartments where the endocytic complexes could reside. To this end, we studied the colocalization of EGF-QDs with early endosomal markers, such as early endosomal antigen 1 (EEA1) and EGF-QDs with lysosomal markers, such as LAMP-1 at *t* = 15, 30, and 45 min, respectively.

**Figure 5:**
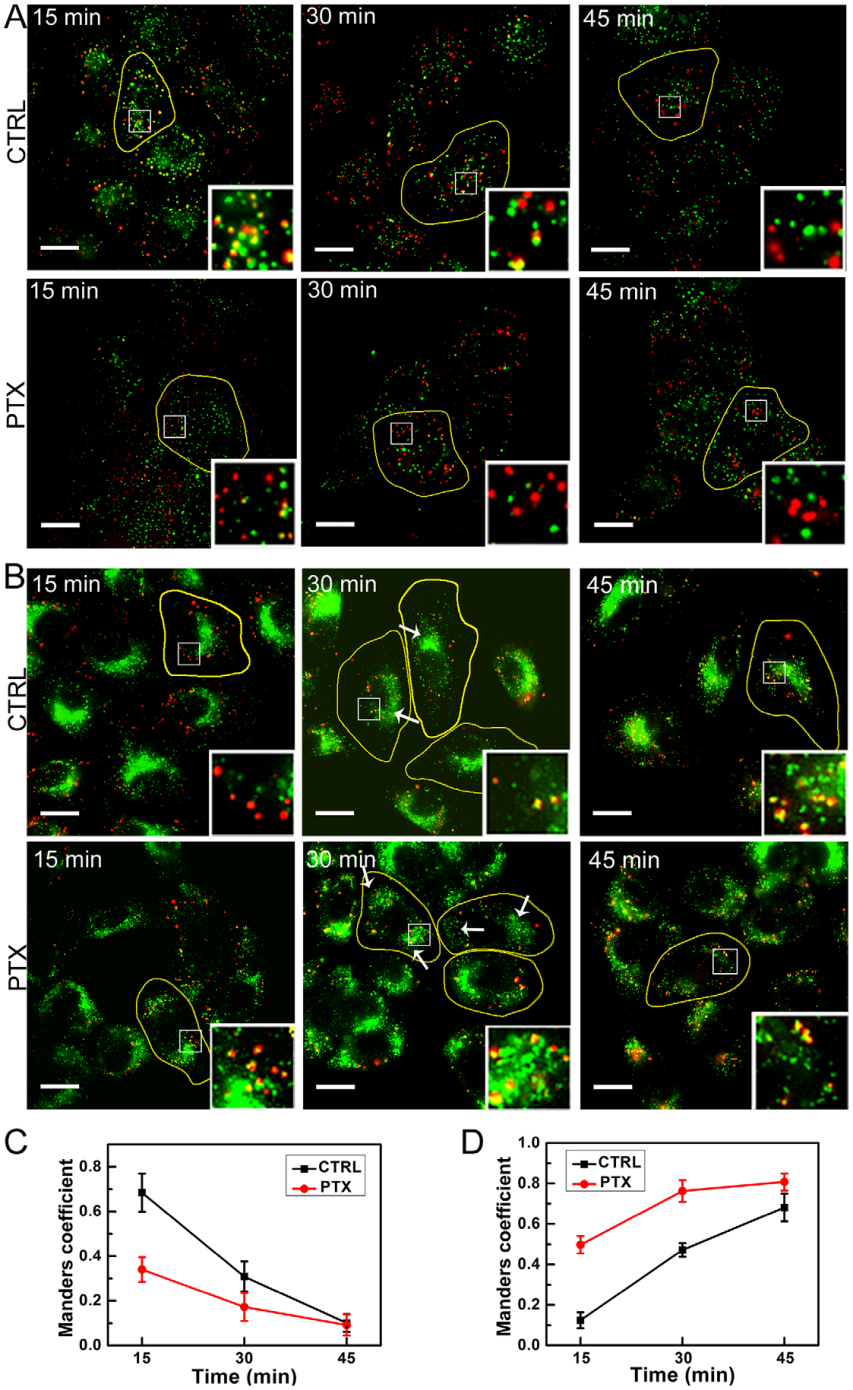
Immuno-colocalization of EGF-QDs with early endosomes and lysosomes. ( A) Intracellular location of EGF-QDs and early endosomes (EEA1) at *t* = 15, 30, and 45 min. The images show the merging of QD (*red*) and EEA1 (*green*) photographs in the same focal plane. Yellow puncta in the merged images indicate colocalization. The boundaries of the cells are marked by thin yellow lines. (B) Intracellular location of EGF-QDs (*red*) and lysosomes (LAMP-1, *green*). From the right panel (30 min), the peripheral distribution of lysosomes is observed in PTX-treated cells, and gathering sites are indicated by arrows in control and PTX-treated cells. For about 30 min, the internalized EGF-QDs began to appear in lysosomes in the control cells, whereas at 15 min time interval, EGF-QDs nearly reached the lysosomes in PTX-treated cells. (C) Colocalizations between EGF-QDs and EEA1 in control and PTX-treated cells were quantified using Manders’ coefficient (M1: red pixels overlapping green). (D) Colocalizations between EGF-QDs and lysosomes were analyzed as described in C. Data are presented as mean 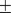 SD; n = 7–10 images. See also Figure S8 for original images. All insets: 4X zoom, all scale bars: 20 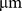. The concentration of PTX used is 100 nM.

The overlying images of EGF-QDs and EEA1 in both control and PTX-treated cells are shown in Figure 5A, whereas those of EGF-QDs and LAMP-1 are shown in Figure 5B (see Figure S8 for original images). The colocalizations were quantified using Manders’ coefficient (Figures 5C and 5D). At *t* = 15 min, the EGF-QDs showed good overlap with EEA1 in the control cells (Figure 5A, upper left), implying that the endocytic EGFRs were inside the early endosomes. However, in PTX-treated cells, less overlapping of EGF-QDs and EEA1 was observed (Figure 5A, lower left). The EGF-QDs that colocalized with the LAMP-1-positive cluster area at the periphery of the cell (Figure 5B, lower left) showed that part of EGFRs had entered the lysosomes. At *t* = 30 min, there were still some EGF-QDs colocalized with EEA1 in the control cells (Figure 5A, upper middle). Meanwhile, the overlap of EGF-QDs and LAMP-1 appeared at the perinuclear area (Figure 5B, upper middle). This result was in line with previous experiments showing that in normal cells, when *t* = 30 min, the EGFRs were transported from early endosomes into lysosomes [41], [58]. By contrast, nearly all of the EGF-QDs had been released from early endosomes (Figure 5A, lower middle) and delivered into lysosomes in the PTX-treated cells (Figure 5B, lower middle) at *t = *30 min. The restricted endocytic transport by PTX was also observed. A great number of EGF-QDs had moved from the periphery toward the cell center in the control cells, whereas the endocytic EGF-QDs were mostly peripherally located in the PTX-treated cells. At *t* = 45 min, in both control and PTX-treated cells, there was no observed colocalization of EGF-QDs with EEA1 (Figure 5A, right), and most of the EGF-QDs were colocalized with LAMP-1 (Figure 5B, right).

Together with the quantitative results shown in Figures 5C and 5D, it is revealed that PTX altered the temporal pattern of endocytic EGF-QDs. The values of Manders’ coefficients in PTX-treated cells at *t* = 15 min were close to those in control cells at *t* = 30 min. The duration of endosomal trafficking (from internalization sites to lysosomes) was about 30 min in control cells, but was only about 15 min in PTX-treated cells. During the first 15 min, the endocytic complexes underwent a fast intracellular sorting process from early endosomes to lysosomes in the presence of PTX. PTX treatment resulted in an altered lysosomal distribution from normal perinuclear to aberrant periphery cytoplasm area, as seen in previous data [29]. As shown in Figure 5B (upper middle), fluorescent signals from the lysosomal marker in the control cells were perinuclear and gathered at one site (*arrow*). The periphery of the cytoplasm was almost completely free of staining. By contrast, the lysosomes in the PTX-treated cells were close to the cell boundaries, where usually two or more gathering sites existed (Figure 5B, lower middle, *arrow*), which could result in higher amount of EGF-QD accumulation in the lysosomes for only 15 min. Hamm-Alvarez et al. also demonstrated that lysosomes are distributed peripherally in the cytoplasm of PTX-treated cells [29]. However, they did not provide the relationship of endocytic events with altered lysosome distribution. We further inferred that the shortened duration of endosomal transport in the PTX-treated cells possibly impairs cellular signaling, as early or late endosomes serve as important platforms during signal transduction [4], [59]. Moreover, considering that the internalized EGFRs in lysosomes are expected to be degraded [6], we supposed that through the fast lysosomal delivery of endocytic receptors, PTX treatment could cause the down-regulation of cell-surface EGFRs. Decrease in numbers of both transferrin receptors and bradykinin receptors was detected on the cell membrane by PTX treatment [28], [29], which supports our view. As the overexpression level of EGFRs directly correlates with many epithelial tumors and is a potential target for therapeutic intervention [34], [60], [61], the down-regulation of membrane EGFR could be a potential anticancer effect by PTX treatment.

**Figure 6:**
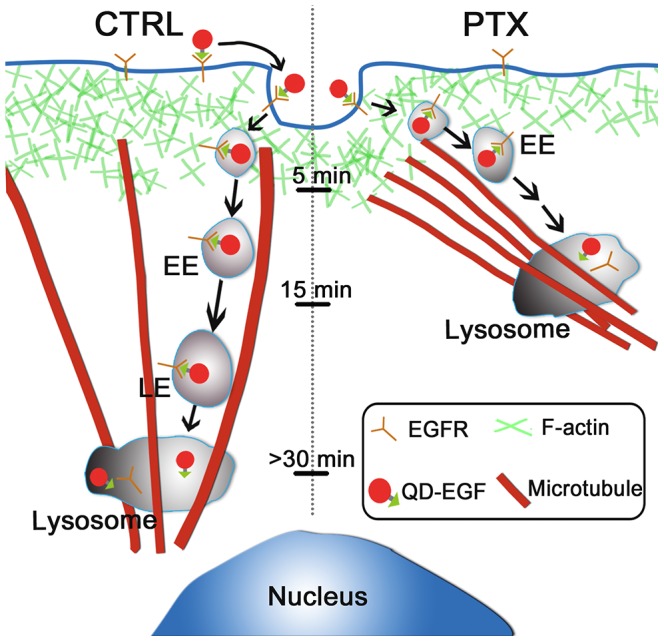
Proposed model for EGFR endocytic trafficking under PTX treatment. The MTs form bundles near the periphery of PTX-treated cells, whereas in control cells, MTs are oriented from an MT organization center near the nucleus. The positioning of lysosomes is associated with MTs via MT-binding proteins, so the lysosomes are anchored near the cell periphery in PTX-treated cells, whereas they are near the nucleus in control cells. As a result, the endocytic EGFRs translocate over a relative short distance from beneath the cell membrane to lysosomes located near the periphery and are degraded (right panel). By contrast, in control cells, most EGFRs translocate over a relatively long distance from beneath the cell membrane to lysosomes located near the nucleus (left panel). The time duration from internalization to lysosomal accumulation is about 15 min in PTX-treated cells, whereas it is over 30 min in control cells. EE, early endosome; LE, late endosome.

Based on our findings, the proposed model for EGFR endocytic trafficking under PTX treatment is shown in Figure 6. The MTs form bundles near the periphery of the PTX-treated cells. On the other hand, the positioning of lysosomes is associated with MTs via MT-binding proteins [62], [63]. Thus, lysosomes are anchored near the cells’ periphery in the PTX-treated cells. As a result, the endocytic EGFRs translocate mainly via super-diffusive mode of motion over a relatively short distance from beneath the cell membrane to the lysosomes located near the periphery, and are then subsequently degraded at the lysosomes (right panel of Figure 6). By contrast, in control cells, most EGFRs translocate over a relatively long distance from beneath the cell membrane to the lysosomes located near the nucleus (left panel of Figure 6). Consequently, the time duration from internalization to lysosomal accumulation is only about 15 min in PTX-treated cells, whereas it is over 30 min in control cells.

In summary, we studied the dynamic characteristics of endocytic trafficking in single cells by tracking all EVs of A549 cells simultaneously with EGF-QDs. We quantified and compared the distinct dynamic behaviors of endocytic trafficking in control and PTX-treated cells. Compared with the control cells, the velocity of directed motion during endocytic trafficking was reduced via suppression of high-speed movements of EVs along MTs in PTX-treated cells. The endocytic trafficking in PTX-treated cells was mainly via super-diffusive mode of motion, whereas in control cells, it was mainly via sub-diffusive mode of motion. In addition, by co-localizing EGF-QDs in early endosomes and lysosomes, we found that the endocytic EGFRs were similarly delivered from early endosomes into lysosomes in both control and PTX-treated cells. However, the lysosomes in PTX-treated cells were close to the cell boundaries, whereas those in the control cells were perinuclear, resulting in most EVs ending near the cell periphery in PTX-treated cells while ending at perinuclear region in control cells. Thus, the duration of endosomal trafficking in PTX-treated cells was only half of that in control cells. These results suggest that PTX, besides having the MT-stabilizing effect as chemotherapeutics, could have other effects on cellular signaling by shortening endosomal trafficking and restricting EGF-QDs to be distributed spatially away from the perinuclear area. The present single-cell study may help us understand the mechanism of the effect of paclitaxel on the treatment of lung cancer. Finally, we should point out that the effects of PTX on endocytic trafficking may be diverse in different cell lines. For example, our preliminary results for human ovarian cancer SKOV-3 cells and breast cancer MCF-7 cells (data not shown) are distinct from those reported here for A549 cells. The detailed studies in other cell lines will be done in future experiments.

## Materials and Methods

### Reagents and Antibodies

Epidermal growth factor biotin conjugate (biotin-EGF), Qdot streptavidin conjugate 655 nm (streptavidin-QD), and PTX were purchased from Invitrogen Corporation. Nocodazole (NOC) and Cytochalasin D (cyto-D) were from Sigma-Aldrich. The rat anti-Tubulin monoclonal, mouse anti-EEA1 monoclonal, and mouse anti-LAMP-1 monoclonal antibodies were from Abcam Coporation. Alexa-488 goat anti-rat IgG conjugate, Alexa-488 goat anti-mouse IgG conjugate, and DAPI were from Invitrogen Corporation. Other chemicals were from Sigma-Aldrich unless otherwise stated.

### Cell Culture and Drug Treatment

Human lung carcinoma A549 cells (ATCC) were maintained in Dulbecco’s modified Eagle medium (DMEM, GIBCO) with 10% fetal bovine serum (FBS, GIBCO) and 1% penicillin-streptomycin (GIBCO) incubated at 37°C with 5% CO_2_. Cells at the log phase were seeded in Petri dishes with poly-L-Lysine-coated glass coverslips on the bottom the day before the experiments were conducted. PTX stock solution was prepared with DMSO at 1.87 mM concentration. The working solution was prepared with fresh DMEM at 100 nM concentration just before the experiments. The final concentration of DMSO in the PTX working solutions was below 0.005%, which was thought to have no harmful effect on the cells. The cells were washed three times with DMEM media and incubated with PTX working solution for 4 h. Then, the PTX medium was removed from the cells. To disrupt microtubules, the cells were incubated at 60 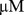 NOC for 30 min. To disrupt actin filaments, the cells were incubated at 20 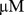 cyto-D for 10 min. NOC and cyto-D were maintained in the medium throughout the experiments.

### EGF and QD Labeling of Cells

The consecutive binding of biotin-EGF and streptavidin-QDs was described previously [16], [36], [37], [38]. Through this labeling method and the biotin-streptavidin system, it was ensured that no free EGF or QD existed in the imaging medium. The number of labeled QDs was about 300 per cell, making the sensitivity of detection extend down to individual QDs. Briefly, cells were washed in cold Hepes medium three times prior to labeling with 10 nM biotin-EGF on ice in Hepes medium for 10 min. After washing, the cells were incubated on ice at 500 pM streptavidin-QDs for 1 min. Subsequently, the cells were washed four times in PBS to remove free biotin-EGF and QDs. Then serum-free, phenol red-free DMEM was added as the imaging medium. The binding was performed at 4°C and internalization was not allowed until the temperature reached 37°C. All the experiments were synchronized by controlling the temperature to obtain the accurate timescale of endocytosis.

### Live Cell Microscopy

Imaging of endocytosis of EGF-QDs was performed with epi-fluorescent microscopy using an inverted Olympus IX70 microscope equipped with a 60X oil objective (1.45 N.A., Olympus) and back-illuminated EMCCD camera (DU-897, Andor Technology). To analyze the quantity of individual signals, a 60X objective with low N.A. (0.7 N.A., Olympus) was used. The microscope was equipped with a CO_2_ incubation system (TOKAI HIT), and the whole course of live cell imaging was performed at 37°C with 5% CO_2_ condition, which is crucial for maintaining the physiological environment of cells. Signals from EGF-QDs were detected with Hg+ lamp using an excitation filter BP530 ± 20 nm and an emission filter (BA590 nm). Image series were recorded at a frame rate of 10 Hz. For a typical image sequence of 30 min, up to 18,000 consecutive frames were obtained with IQ software (Andor Technology). DIC optics (Olympus) was used to obtain cell body images before and after the fluorescence imaging. The cells in mitosis phase were eliminated.

### EGF-QD Tracking

To study the movement of EGF-QD containing endocytic vesicles (EVs) in the cytoplasm, we performed single-particle tracking with the aid of the ImageJ Particle Tracker Plugin software with a spatial resolution of 10 nm to 30 nm developed by Sbalzarini and Koumoutsakos [64]. For each video clip, individual EVs were detected by adjusting parameters for radius, cutoff, and percentile to maximize capture of the greatest number of QDs. The parameters of linking range and displacement were adjusted to link the detected particles between frames. The linking step was checked optically for possible inappropriate linking adjustments. The EGF-QDs imaged during the endocytic trafficking aggregated, fully reducing the single-QD blinking. The software was able to detect the fast movements at 10 Hz. The information of the detected EVs was saved as text files. All individual trajectories visually detected were included in our analysis database and trajectories longer than 50 frames were selected for further analysis. All the EGF-QD positions inside a live single cell in each frame were saved in text files and analyzed using user-defined program in Matlab (MathWorks).

### EGF-QD Intensity Analysis in Single Cells

The dynamic behavior of endocytosis was observed during the first 5 min after the cells were heated up to 37°C. This result was characterized by the average intensity of all the visible EGF-QDs in a single cell. In each frame, the intensities of all puncta (single or aggregated QDs) in one cell were collected using the ImageJ Plugin and then averaged. The plots of the averaged intensities were normalized to the initial value.

### Endocytic Ratio Calculation

The dynamic behavior of endocytic trafficking was characterized by the parameter called endocytic ratio, defined as the ratio of the average radius of the cell (distance of points on cell boundaries to the cell center) to the average distance of all the EGF-QDs to the cell center in each frame. The boundary of the cell was discernible in the DIC images as distinct cleft-like contours, whereas the center of the cell was the nucleus, which is visible as ovoid regions surrounded by high-contrast vesicular structures. The boundary and the center were manually selected in DIC images using software ImageJ. Ideally, the value of endocytic ratio should increase when the EVs move toward the center of cells. However, it should be equal to 1 if all the EVs are at the cell boundary. Most EVs moved from the periphery toward the center of the cell after receptor internalization on the cell membrane, so the value of endocytic ratio increases from the value above 1 during endocytic trafficking.

### Trajectory Classification and Analysis in Single Cells

To quantify the dynamics of EGF-QDs in single cells, the trajectories were analyzed by mean square displacement (MSD) and directional persistence, as done previously [45]. For each time point *i* of the trajectory, the directional persistence of the trajectory was calculated as
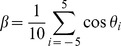
where 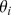 denotes the change of angle between adjacent steps of the trajectory and 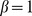 corresponds to the perfect unidirectional motion. The local MSD function was calculated as



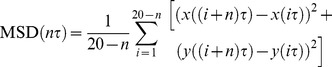
where 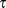 is the acquisition time, *n = *1,…, 10. MSD was fitted by the power law 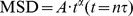. 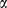 indicates the non-linear relationship of MSD with time, which carries information about the local motion modes [53], [54]. When 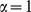, the particle undergoes free Brownian motion (e.g., pure random walk, free diffusion); when 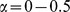, the constrained motion (e.g., near immobility); when 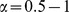, the sub-diffusion (e.g., diffusion within a confined area); when 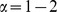, the super-diffusion (e.g., diffusion overlaid with deterministic motion); and when 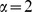, the ideal directed motion. The sample size for dynamics analysis is shown in Table S1.

The point of the trajectories is defined as “directed state” when two criteria are fulfilled: 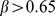 and 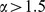, to achieve sufficient discrimination of directed motion. If the consecutive points in directed state are longer than or equal to 5 frames and their displacements in 0.5 s are more than 1 pixel (267 nm), they would be extracted as one directed segment. This method would help us obtain the directed motion with high accuracy and disregard the transient directed movements shorter than five frames. To consider the random errors associated with the vesicle position measurement in each frame, we computed each instantaneous velocity component using 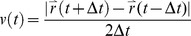, where 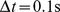. The velocities were analyzed statistically with a 5 min interval for each observation using a two-step method: first, the mean values of velocities in all the directed motion in each cell were calculated every 5 min. Each point of the directed motion was considered as one sample. Second, the mean velocities for all cells for every 5 min were averaged.

For our method verification, we used support vector machine (SVM) classifier, which was developed by Helmuth et al. [65]. The trajectories were manually segmented to generate the sample data containing directed motion and non-directed motion. Next, the classifier was trained to distinguish the directed motion. After optimization of the classifier parameters, SVM was used to process the raw data and to calculate velocities.

The total motility was studied by analyzing the time-resolved MSD of the whole trajectories. Using the above fitted parameters 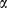, we classified the movements of EVs into three modes of motion: constrained motion (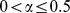), sub-diffusion (), and super-diffusion (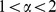) (Figure S6). Objects larger than 20 nm could not diffuse freely through the crowded cytoplasm [55]. In our experiments, the endocytic vesicles that contained several EGFR-EGF-QD complexes (a single streptavidin QD has a diameter of about 20 nm) appeared significantly larger than 20 nm; thus, the free Brownian motion could be highly restricted. The diffusion constant *D* was determined by fitting the initial 5 points of the MSD curve with 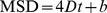
(dashed lines in Figure S6) [37]. The statistical calculation for 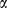 and *D* were obtained using the same method used to calculate velocities.

### Immuno-colocalization and Quantity Analysis

Cells in the log phase were seeded on glass coverslips and incubated overnight. The 100 nM PTX treatment was done for 4 h, with consecutive binding of EGF-QD. Next, cells were fixed with 4% paraformaldehyde in PBS at time points *t* = 15, 30, 45 min after internalization, whereas *t* = 60 min for MT immunostaining experiments. Then, cells were permeabilized in 0.2% Triton X-100 in PBS and blocked with 1% BSA for 1 h at room temperature before the application of antibodies against target proteins. The cells were immunostained by the following primary antibodies: rat anti-Tubulin (1∶500) monoclonal, mouse anti-EEA1 (1∶100) monoclonal, mouse anti-LAMP-1 (1∶100) monoclonal, and the secondary antibody-fluorescent dye conjugates Alexa-488 goat anti-rat IgG (1∶300) and anti-mouse IgG (1∶300). Chromosomes were stained with DAPI. Fluorescence images were obtained at the same focal plane as EGF-QDs using an inverted Olympus IX70 epi-fluorescence microscopy. The images were colored and merged with ImageJ software.

The quantitative colocalization analysis was performed with ImageJ and JACoP Plugin [66] to determine Manders’ coefficient (M1, for red pixels overlapping green pixels). M1 is defined as the ratio of the ‘total intensities of pixels from the red image (QD puncta), of which the intensity in the green channel (EEA1 or LAMP-1) is above the threshold’ to the ‘total intensity in the red channel’. The Manders coefficients for all images at each time interval were averaged and plotted against time.

## Supporting Information

Figure S1 **The normalized average intensity of punctate fluorescence acquired using a 0.7 N.A. lens in control cells.** The experimental conditions were the same as those in Figure 1B. Increasing the intensity of fluorescence indicated the fusion of QDs in endosomes, which was the same as using 1.45 N.A. lens (Figure 1B, CTRL).
(TIF)

Figure S2
**Number of EGF-QDs tested at different focus planes.** The depth of field of the objective was less than 1 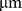 and the thickness of the A549 cell was about 4 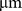 to 5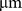, so only one layer of the cells could be continually visualized real time. We selected the focus plane along the z-axis equal to 1 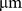 from the surface of the glass, and quantified the number of EGF-QDs in focus compared with the out of focus at different time intervals. (A) Images of EGF-QDs in an A549 cell at the same time (*t* = 1 min), but in different focus planes: *z = *1, 2, 3.5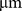. The boundary of the cell was marked by a white line. Scale bar: 10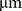. (B) Histograms showing the number of QDs in focus (*z* = 1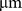) and out of focus during different time intervals. The visible EGF-QDs in focus were dominant for every 5 min time interval window; thus, the out of focus EGF-QDs that were neglected would not affect the results. Data were from more than 3 cells; mean ± SE.(TIF)

Figure S3
**Similar endocytic patterns in PTX-treated and NOC-treated cells** (A) The time-lapse images of endocytic traffic in NOC-treated cells at *t* = 0, 5, 30, 60 min interval (time resolution: 100 ms). The fluorescent image of the QDs was overlaid with the bright-field DIC image of the cell. (B) The endocytic ratio as the function of time in both PTX-treated (same as in Figure 2C) and NOC-treated cells. The cells were treated with 100 nM PTX for 4 h or with 60 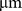 of NOC for 30 min prior to the experiments. The thick lines indicate the average values of the adjacent 20 points along the original lines.(TIF)

Figure S4 **Comparison of directed motion velocities obtained from our method with those confirmed by Support Vector Machines (SVM).** The raw trajectory data of one control cell and one PTX-treated cell’s directed motion were extracted with the use of our method and the SVM, seperately. Similar results in velocities at different time intervals verified the reliability of our method.
(TIF)

Figure S5 **Typical probability distributions of velocities during** ***t*** **= 35 min to 40 min interval.** Inset: cumulative distributions for control (*black*) and PTX-treated (*red*) data.
(TIF)

Figure S6 **MSD versus time of trajectory points in three modes of motion shown in** **Figure 4A** **.** The MSD was calculated over 20 frames, and the non-linear relation was fitted using the first 10 points. The dashed lines in different colors are the linear fitting of MSD using the first five points. The slopes of the dashed lines correspond to the diffusion coefficient *D*.
(TIF)

Figure S7
**Probability distributions of 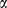 during** ***t*** **= 45 min to 50 min interval in control and PTX-treated cells.** The solid line indicates the difference in value between the two cases in each bin.(TIF)

Figure S8
**Original images of bright field, EGF-QDs, early endosomes, and lysosomes shown in** **Figure 5** **.** Cells were fixed at time *t* = 15, 30, 45 min after EGF-QD internalization, were permeabilized and labeled with early endosome marker (EEA1) and lysosome marker (LAMP-1). A) The bright field, QD, and EEA1 images in control and PTX-treated cells corresponding to Figure 5A. B) The bright field, QD, and lysosome images corresponding to Figure 5B. All scale bars, 20 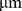.(TIF)

Table S1 **Sample size for dynamic analysis.**
(DOC)

Movie S1
**The fusion of EVs in PTX-treated cells in the boxed area shown in** **Figure 1A** **.** Images were captured on time-lapse epifluorescence microscopy immediately after the cells were heated up to 37°C for 5 min. Image size: 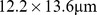.(AVI)

Movie S2
**Live imaging of endocytic trafficking of EVs in control cells shown in** **Figure 2A** **.** The fluorescent images of the EGF-QDs were overlaid with the bright-field DIC image of the cell. Images were acquired on time-lapse epifluorescence microscopy equipped with a CO_2_ incubation system at 1 s interval for 60 min. Image size: 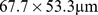 (time stamp in min: sec).(AVI)

Movie S3
**Live imaging of endocytic trafficking of EVs in PTX-treated cells shown in** **Figure 2A** **.** The images were obtained and processed as described in Movie S2. Image size: 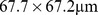 (time stamp in min: sec).(AVI)

Movie S4
**Tracking the movement of EV shown in** **Figure 3A** **.** The EGF-QD trajectroy with the path marked by cyan line was produced using ImageJ Particle Tracker Plugin software. Image size: 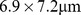.(AVI)
