# Supplementary figures and images for: Effects of Paclitaxel on EGFR Endocytic Trafficking Revealed Using Quantum Dot Tracking in Single Cells
Source: PLoS One. 2012 Sep 20;7(9):e45465. doi: 10.1371/journal.pone.0045465 (PMC3447934; doi:10.1371/journal.pone.0045465)

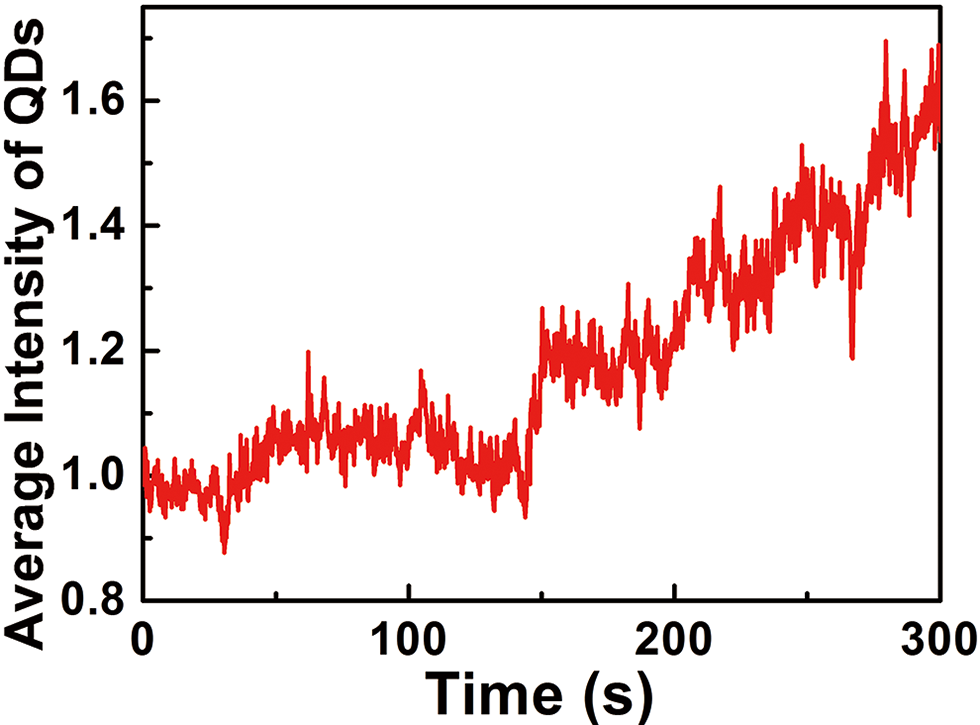

Supplement: Figure S1 — The normalized average intensity of punctate fluorescence acquired using a 0.7 N.A. lens in control cells. The experimental conditions were the same as those in Figure 1B. Increasing the intensity of fluorescence indicated the fusion of QDs in endosomes, which was the same as using 1.45 N.A. lens (Figure 1B, CTRL). (TIF) [file pone.0045465.s001.tif]

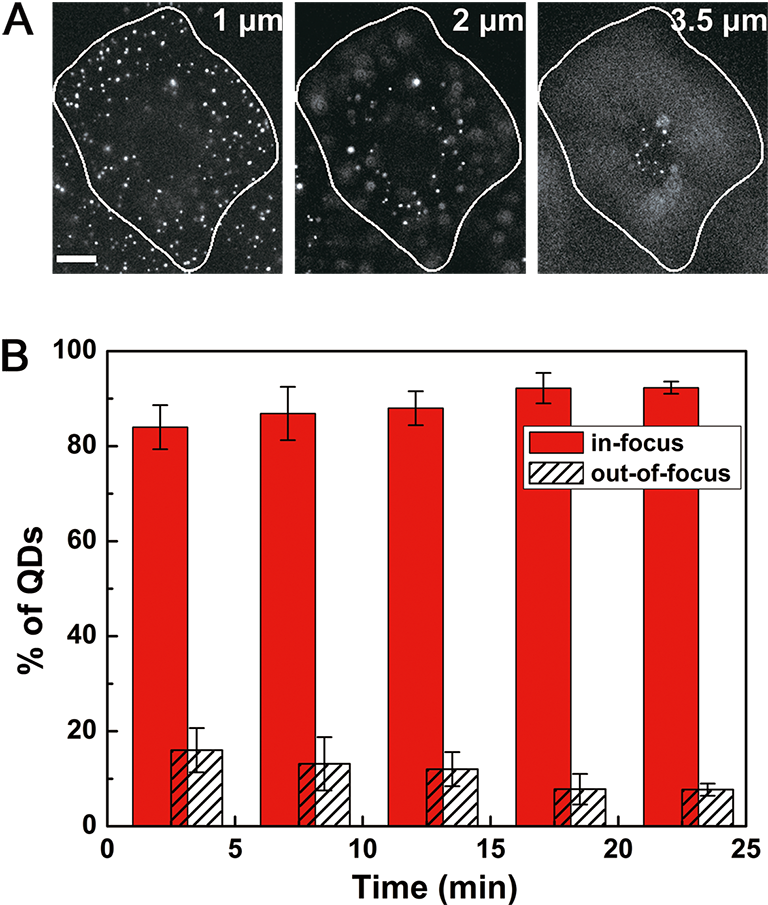

Supplement: Figure S2 — Number of EGF-QDs tested at different focus planes. The depth of field of the objective was less than 1 and the thickness of the A549 cell was about 4 to 5, so only one layer of the cells could be continually visualized real time. We selected the focus plane along the z-axis equal to 1 from the surface of the glass, and quantified the number of EGF-QDs in focus compared with the out of focus at different time intervals. (A) Images of EGF-QDs in an A549 cell at the same time (t = 1 min), but in different focus planes: z = 1, 2, 3.5. The boundary of the cell was marked by a white line. Scale bar: 10. (B) Histograms showing the number of QDs in focus (z = 1) and out of focus during different time intervals. The visible EGF-QDs in focus were dominant for every 5 min time interval window; thus, the out of focus EGF-QDs that were neglected would not affect the results. Data were from more than 3 cells; mean ± SE. (TIF) [file pone.0045465.s002.tif]

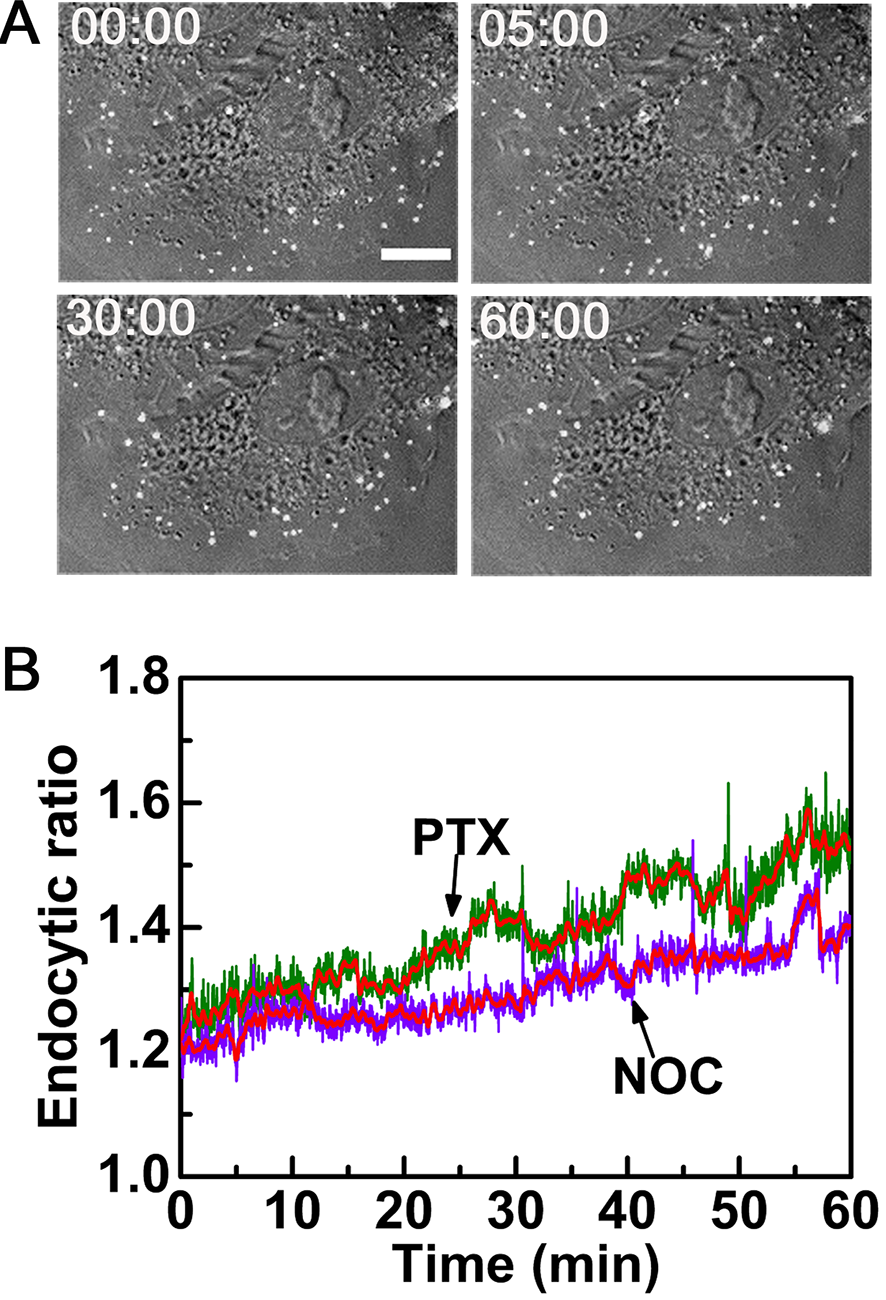

Supplement: Figure S3 — Similar endocytic patterns in PTX-treated and NOC-treated cells (A) The time-lapse images of endocytic traffic in NOC-treated cells at t = 0, 5, 30, 60 min interval (time resolution: 100 ms). The fluorescent image of the QDs was overlaid with the bright-field DIC image of the cell. (B) The endocytic ratio as the function of time in both PTX-treated (same as in Figure 2C) and NOC-treated cells. The cells were treated with 100 nM PTX for 4 h or with 60 of NOC for 30 min prior to the experiments. The thick lines indicate the average values of the adjacent 20 points along the original lines. (TIF) [file pone.0045465.s003.tif]

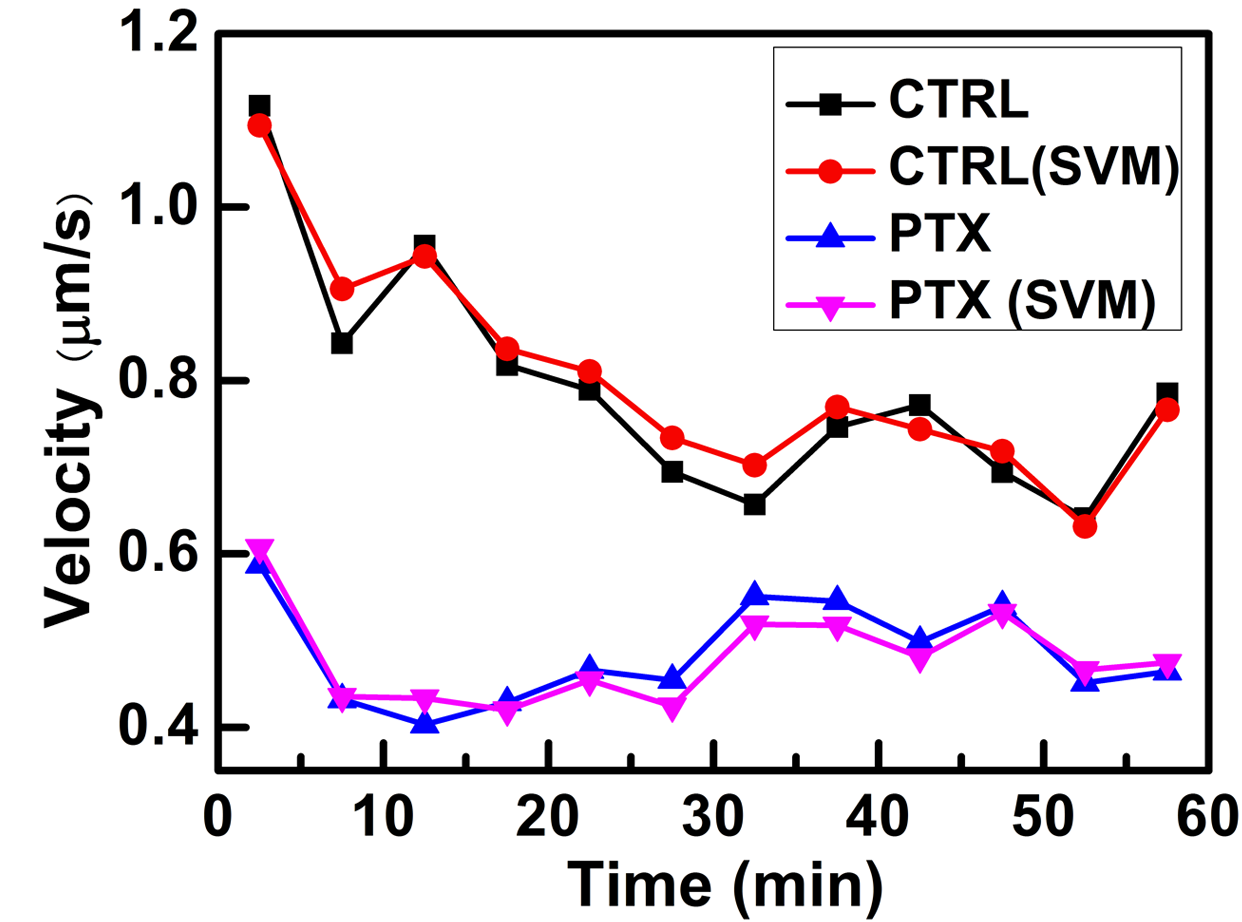

Supplement: Figure S4 — Comparison of directed motion velocities obtained from our method with those confirmed by Support Vector Machines (SVM). The raw trajectory data of one control cell and one PTX-treated cell’s directed motion were extracted with the use of our method and the SVM, seperately. Similar results in velocities at different time intervals verified the reliability of our method. (TIF) [file pone.0045465.s004.tif]

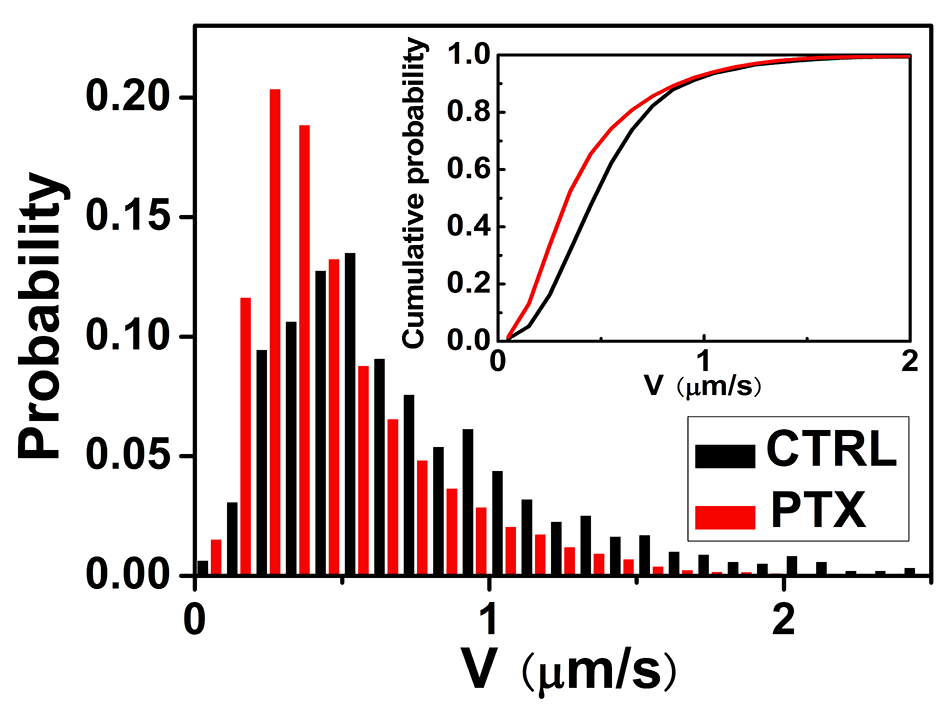

Supplement: Figure S5 — Typical probability distributions of velocities during t = 35 min to 40 min interval. Inset: cumulative distributions for control (black) and PTX-treated (red) data. (TIF) [file pone.0045465.s005.tif]

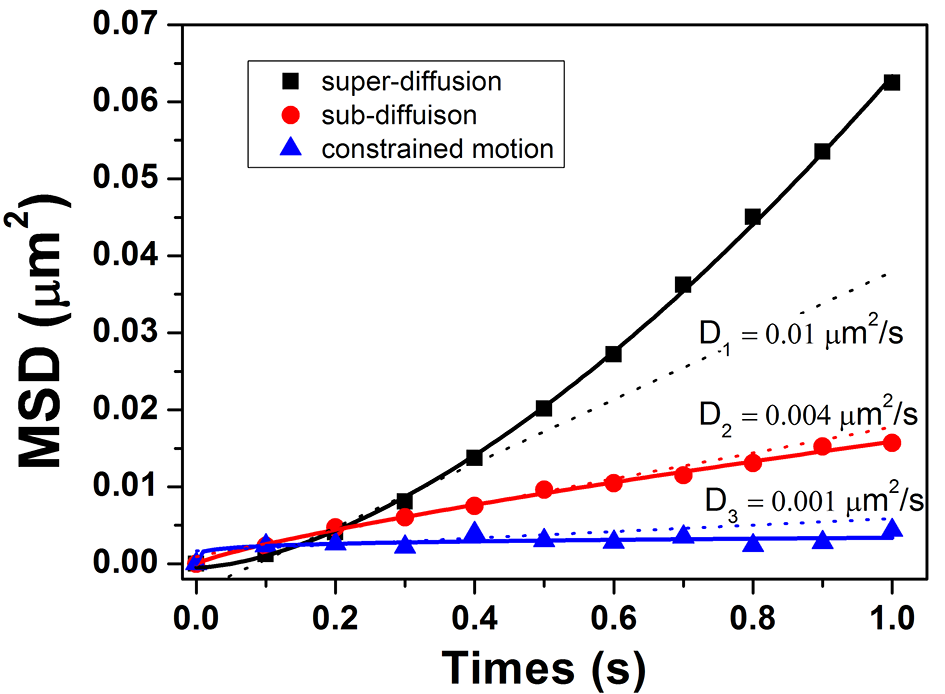

Supplement: Figure S6 — MSD versus time of trajectory points in three modes of motion shown in Figure 4A . The MSD was calculated over 20 frames, and the non-linear relation was fitted using the first 10 points. The dashed lines in different colors are the linear fitting of MSD using the first five points. The slopes of the dashed lines correspond to the diffusion coefficient D. (TIF) [file pone.0045465.s006.tif]

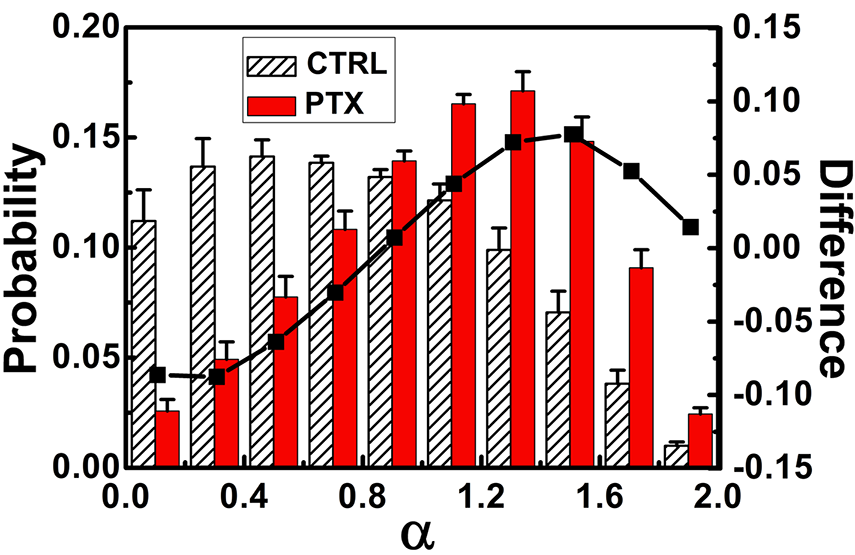

Supplement: Figure S7 — Probability distributions of during t = 45 min to 50 min interval in control and PTX-treated cells. The solid line indicates the difference in value between the two cases in each bin. (TIF) [file pone.0045465.s007.tif]

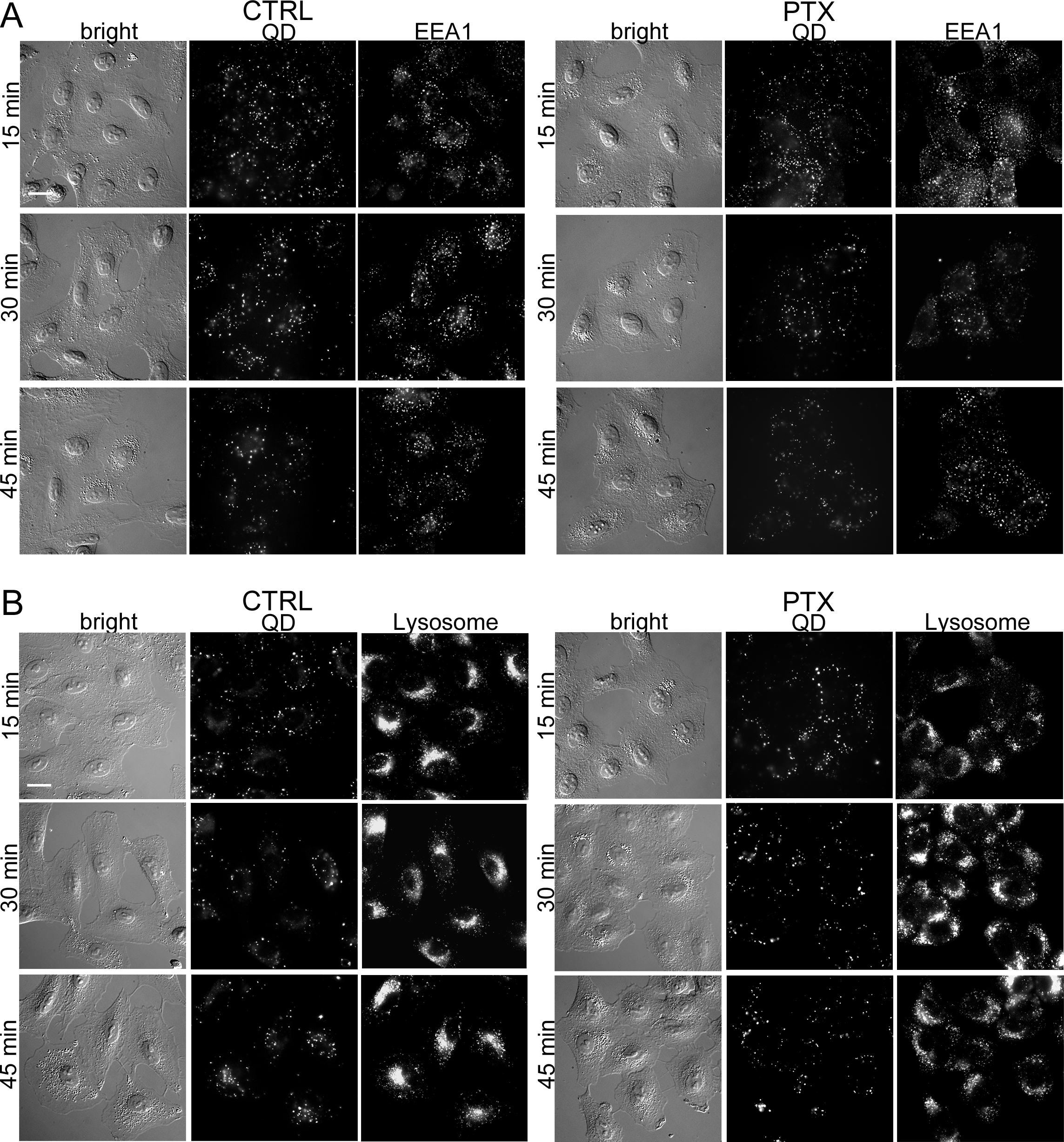

Supplement: Figure S8 — Original images of bright field, EGF-QDs, early endosomes, and lysosomes shown in Figure 5 . Cells were fixed at time t = 15, 30, 45 min after EGF-QD internalization, were permeabilized and labeled with early endosome marker (EEA1) and lysosome marker (LAMP-1). A) The bright field, QD, and EEA1 images in control and PTX-treated cells corresponding to Figure 5A. B) The bright field, QD, and lysosome images corresponding to Figure 5B. All scale bars, 20 . (TIF) [file pone.0045465.s008.tif]
