# Supplementary material for: Effects of Paclitaxel on EGFR Endocytic Trafficking Revealed Using Quantum Dot Tracking in Single Cells
Source: PLoS One. 2012 Sep 20;7(9):e45465. doi: 10.1371/journal.pone.0045465 (PMC3447934; doi:10.1371/journal.pone.0045465)
Supplement: Table S1 — Sample size for dynamic analysis. (DOC) [file pone.0045465.s009.doc]

Table S1. Sample size for trajectory analysis

|  | Cells | Tracks per cell | Directed motion segments per cell | Velocity sample per cell | Directed motion per cell (%, in time) | Mean velocity (μm/s) | Mean  run length (μm) |
| --- | --- | --- | --- | --- | --- | --- | --- |
| CTRL | 6 | 4825 ± 487 | 1056 ± 184 | 15603 ± 2865 | 1.28 ± 0.15 | 0.72 ± 0.03 | 1.02 ± 0.03 |
| PTX | 8 | 2474 ± 193 | 2486 ± 359 | 47205 ± 7532 | 2.99 ± 0.17 | 0.51 ± 0.01 | 0.94 ± 0.02 |
| NOC | 3 | 4704 ± 893 | 12 ± 1 |  | 0.022 ± 0.003 |  |  |
